# Consumer attitudes and concerns with bioplastics use: An international study

**DOI:** 10.1371/journal.pone.0266918

**Published:** 2022-04-27

**Authors:** Walter Leal Filho, Jelena Barbir, Ismaila Rimi Abubakar, Arminda Paço, Zaneta Stasiskiene, Marie Hornbogen, Maren Theresa Christin Fendt, Viktoria Voronova, Marija Klõga

**Affiliations:** 1 Hamburg University of Applied Sciences, Faculty of Life Sciences, Research and Transfer Centre “Sustainable Development & Climate Change Management” (FTZ-NK), Hamburg, Germany; 2 College of Architecture and Planning, Imam Abdulrahman Bin Faisal University (formerly, University of Dammam), Dammam, Saudi Arabia; 3 University of Beira Interior, Department of Management and Economics, Núcleo de Estudos em Ciências Empresariais (NECEUBI), Rua Marquês d’Ávila e Bolama, Covilhã, Portugal; 4 Kaunas University of Technology, Institute of Environmental Engineering, Kaunas, Lithuania; 5 Tallinn University of Technology, Department of Civil Engineering and Architecture, Tallinn, Estonia; Karl-Franzens-Universitat Graz, AUSTRIA

## Abstract

The world production of plastic exceeded 360 million tonnes in 2020 alone, a considerable amount of which is not properly disposed of. The significant pressures and damages posed by conventional plastic to human and environmental health suggest that alternatives are urgently needed. One of them is “bioplastic”, which is defined as bio-based plastic that is (or not) biodegradable. This paper reports on a study on the perceptions of bioplastics among consumers in 42 countries to identify their levels of information and concerns. The results suggest that most respondents have positive expectations regarding the future of bioplastics to replace conventional plastics fully or partially, especially for food containers, kitchenware, and boxes and bags for packaging. They also reported that the low costs and increased availability of bioplastic products on the market are likely to be the main drivers for their wide-scale adoption. However, many participants are unsure whether they would buy bio-based and biodegradable products if they are expensive. Overall, whereas a rather positive attitude to bioplastics has been identified, greater efforts are needed to address the many information needs of consumers towards upscaling the adoption of bioplastics. Relevant policies are therefore needed to encourage investments in the large-scale manufacture and market uptake of bioplastics. The paper reports on an initial study of consumer behavior, in a sample of countries spread across all geographical regions.

## 1. The problems posed by conventional plastics

Plastics have become one of the most widespread materials used globally, and global production has on average increased by about 9% per year since 1950, reaching 367 million tonnes per annum in 2020 [1]. According to [2], this rapid growth has been driven by two underlying trends:

- the continued growth in consumer demand since plastic is used in a wide range of home and commercial products and
- the low costs associated with plastic production, which makes it very attractive

There are many varieties of plastic being used today. The main ones, according to [3] are:

- Polyethylene terephthalate (PET)—of which plastic bottles, fast food containers, plates, and cups are made;
- High-Density Polyethylene (HDPE)—used for the manufacturing of coloured plastic bottles, fabric softener, engine oil;
- Polyvinyl Chloride (PVC)–used for bottles and glass dish detergents, plastic mats;
- Low-Density Polyethylene (LDPE)—of which juice bottles, mustard cans, beer bottles, and other packaging systems are made;
- Polypropylene (PP)—used for bottles of syrups, yogurt jars, etc.;
- Polystyrene (PS)—of which egg cartons, plastic cutlery, Petri dishes, plastic tubes, etc.;

The combination of the two key elements: high demand due to a variety of applications, and low costs means that plastic is a highly successful product. The downside is that current plastic production is heavily dependent on fossil fuels [4], and the fact that most plastic materials are not biodegradable has led to a growth in the production and use of bioplastic.

Unfortunately, there is a faulty practice in many countries when using the term ‘bioplastics’ for two different things. Firstly, there are bio-based plastics (plastics made at least partly from the biological matter) and secondly, there are biodegradable plastics (plastics that can be completely broken down by microbes in a reasonable timeframe, given specific conditions). However, not all bio-based plastics are biodegradable. Also, even biodegradable plastics might not fully biodegrade in unfavorable environments. Therefore, many issues remain unsolved [2, 5, 6]. Considering international approach within the study, the term “bioplastics” is used for easier evaluation of consumer attitudes.

Another very important issue is that the ‘end of life in the plastic chain does not mean ‘end of the impact’. Because plastic materials persist and pollute long after their intended use, it has become clear that there is no such thing as an ‘end of life’ for plastics. Regardless of the disposal method, plastics may pose a significant threat to the environment and the climate when they reach the waste phase of their life cycles. This phase is when a high proportion of the remaining plastic waste ends up in the environment, in dumping sites, oceans, and other waterways, scattered across natural and human landscapes worldwide.

Also, it should be highlighted that due to the increase in the total manufacturing of plastics, microplastics have become a serious problem. It is necessary to understand microplastic’s prospective sources and sinks, the process by which its distribution is affected, and their uptake and exchange in ecosystems to understand the potential ecological harm done by microplastics. Accordingly, it is estimated that there are more than 5 trillion plastic particles in the world’s surface waters [7]. About 12.7 million metric tons of plastic waste are estimated to enter the ocean each year [8]. Land-based sources include tourism, sewage, and illegal or poorly managed landfills, due to inadequate waste management [2, 9]. Most of the plastic litter arriving in the oceans has been produced, used, and often disposed of on land [8, 10]. According to [11], the microplastics types most commonly observed in the marine environment were PP, LDPE, HDPE, and PET.

It is estimated that approximately 80 percent of ocean plastics come from land-based sources, and the remaining 20 percent are from marine sources [12]. Of the 20 percent from marine sources, it is estimated that around half arises from fishing fleets (such as nets, lines, and abandoned vessels). Figures from the United Nations Environment Programme (UNEP) suggest that abandoned, lost, or discarded fishing gear contributes to approximately 10 percent of the total ocean plastics [13]. Plastic flows depend on the proximity of urban activities, shore, and coastal uses, wind, and currents. Plastic debris is also carried to the sea by rivers, which flow into the sea after passing through densely populated areas [4]. A study by [10] indicated that between 1.15 and 2.41 million tonnes of plastic currently flow from the global riverine system into the oceans every year. The top 20 polluting rivers accounted for two-thirds (67%) of the global annual river input. Most of the river plastic originates from Asia, representing 86 percent of the global total. Africa follows Asia at 7.8 percent and South America at 4.8 percent. Central & North America, Europe, and the Australia-Pacific region collectively account for just over one percent of the world total [10, 14].

## 2. Literature review

### 2.1 Bioplastics and their use

Bioplastics can be defined as bioplastic that is either bio-based or biodegradable or has both of these properties [15]. Bio-based plastics are derived from plant-based biomass or organic material. In contrast, biodegradable plastic refers to the environmental fate of the bioplastic, which suggests that the plastic will break down within a certain timeframe while leaving no toxic residues behind [16].

Bioplastics are considered to be sustainable, environmentally friendly alternatives to conventional plastics. With the growing demand and use of plastics in the industry, many have turned to petroleum-based plastics. However, these are resistant to biodegradation, and the burning of such plastic releases toxic gases, which are harmful to animals, plants, and their natural environment. In this way, biopolymers are more advantageous due to their ability to degrade easily and can be considered a safer alternative to normal plastics [17].

The production of conventional plastics requires a large amount of energy and petroleum, which is becoming scarce. However, bioplastic production involves low energy consumption and may reduce oil consumption in the process. Furthermore, the production emits low carbon and can be carbon neutral due to the absorption of CO_2_ by the biomass components. Additionally, using more natural substances diminishes the need for toxic substances that were previously used to produce conventional plastic [18].

However, the production of bioplastics remains limited due to the high costs involved. This challenge is mainly attributed to the inability to mass-produce, and the cost is 2–3 times higher than conventional plastic. Furthermore, countries lack the technology needed to produce such products. In other instances, the lack of consumer awareness has decreased the use of bioplastics. Therefore, increasing the promotion of these products will greatly increase the demand [18].

Bioplastics can be broken down into three main types based on the material they are made up of. The first group is the bio-based or partly bio-based bioplastics that are not biodegradable such as PP, PET, and PP and bio-based technical performance polymers. The second category consists of bioplastics that are bio-based and biodegradable, which include PBS and PLA. Lastly, there are biodegradable bioplastics that are derived from fossil resources [15].

Bio-based and non-biodegradable are the most common type of bioplastics. These are produced using natural or renewable feedstock and are made using the same pathways, equipment, and technology as their conventional counterparts [16]. They can also be called drop-in or bio-blends. Sometimes, they may contain starch or small quantities of biodegradable material to aid in the fragmentation process; however, the overall product is non-biodegradable ‘[19]. The difference between these bioplastics and their conventional counterparts is seen in the price and environmental friendliness [16]. Bioplastics are more expensive due to the low investment, lower processing capacity, and the cost of raw material. Furthermore, bio-based bioplastics are more environmentally friendly, as the carbon dioxide produced during processing can be captured and used by plants, thus providing more raw materials, whereas this is not possible for conventional plastics [16, 20].

The second type of bioplastics is both bio-based and biodegradable. It may be produced from plant-based materials, by-products of microbial fermentation, and animal-derived polymers. In doing so, the life cycle of biomass is mimicked, and carbon dioxide and water are released while preventing the use of fossil resources [15]. Plant biomass used for this category includes the raw materials from pineapple, barley, starch, wheat, hemp, oat, and flute. These materials are used to extract thermoplastic starch, lignins, rubber, and cellulose, which are needed to produce the required bioplastic [21]. By-products of microbial fermentation are used to produce polyester bioplastics. These are storage polymers produced by microbes using various enzymatic processes to aid in their survival upon stimulation from different stressors and nutrients. However, this method is not commonly used as it has a high production cost and requires more optimization [22]. For animal-derived bioplastics, materials such as chitin, wool, fats, and gelatin are used. In more recent times, collagen has been used to produce bioplastic to reduce waste from meat production [16]. Fossil-based biodegradable bioplastics are made from fossil resources that have a degree of biodegradability. However, hydrophilicity, reactivity, and stability determine the biodegradability and molecular weight of the raw materials used, which is not dependent on their origin. Consequently, these products are less favored in comparison to bio-based bioplastics [23].

In present times, the bioplastics market accounts for the production of nearly 320 million tonnes of plastic annually. However, with the growing demand for these sustainable products, the markets grow from 20 to 100 percent per annum. This situation is facilitated by advancement in research, technological creations, and environmental awareness. As a result, there is a growing demand for these products in various sectors, including packaging, food, textile, automobile, and agriculture. In the year 2016, packaging accounts for the greatest use of such products at nearly 40 percent, followed by consumer goods that accounted for 22 percent of bioplastic demand [24] and the number of bioplastics produced around the world from 2015–2019 has projected an increased production for the years 2020–2023.

### 2.2 Factors influencing consumer attitudes on bioplastics

To make new sustainability schemes, technologies, and products such as bioplastics effective, they need to be accepted by the people, as the major stakeholder in achieving environmental sustainability. People engage in pro-environmental behaviors such as using bioplastics when they have (a) the right attitude, like a concern for the environment or a belief that their action can help lower plastic’s carbon footprint, and (b) the ability to translate the attitude into behavior such as purchasing bioplastic products [25]. However, adopting such green products can be difficult because of people’s different levels of awareness of and attitudes toward the environmental impacts of their consumptions. Lack of awareness and public reluctance can be observed in different types of sustainability initiatives such as circular economy approaches [26], initiatives for disaster risk reduction [27], treated wastewater reuse [28], and green technologies and products [29].

The literature indicates that environmental concerns, beliefs, emotions (feeling of doing something good), product shelf life and price, and trust in the safety of the product are the major factors influencing consumer attitudes towards using bio-based plastics [25, 30–32]. For example, study [25] found that guilt was the most significant factor influencing respondents’ willingness to pay more for bio-based plastic products. In a study of factors that influence customer intentions to buy bio-based products in six European countries, the authors [32] found that the intention to purchase bio-based brands is enhanced by personal environmental norms, such as associating a bio-based product with eco-friendliness as well as whether a green brand is a global or private label. In addition, the study participants indicated their willingness to green purchase only products that consisted of 100% bio-based materials.

This paper [30] investigated consumer attitudes towards bio-based packaging in France, Germany, and the USA. The study reported that the respondents focused principally on end-of-life packaging attributes such as biodegradability (50–60%) and values for reuse (43–55%) and recycling (42–67%). Conversely, respondents were less concerned about whether packages are made from renewable resources (10–13%) and have a low carbon footprint in production and transportation (8–18%). The authors conclude that even in developed countries, there is less awareness about the environmental friendliness of bio-based packaging, and consumers reject the idea of using land to grow raw material for packaging.

This study [29] investigated the consumers’ emotional assessments of and rational beliefs about green packaging in Norway. The study found that emotions (perceived benefits of eco-friendly packaging) are the key driver in purchasing plant-based beverage containers rather than the rational assessments of its ecological benefits. Thus, affective and cognitive processes play a significant role in influencing the consumers’ purchase of green packaging products.

In Germany, [33] explored user preferences for outdoor sporting equipment produced from bioplastics. The surveyed respondents showed positive attitudes toward sports shoes and drink bottles if they can help reduce carbon emission, are made from raw materials cultivated locally (belief in one’s nation and supporting local economy), and are safe products with comparatively low prices. The authors deduced that bioplastics have a promising future if potential users understand their quality and ecological friendliness.

## 3. Methods

A questionnaire was used to explore the level of awareness and attitudes about bioplastics, using a sample of individuals from a set of 42 countries located mostly in Europe and Asia. The method of data collection was a survey, taking the form of a self-administered questionnaire, and consisting mainly of closed questions that covered the following sections: (i) demographics (country of residence, gender, age, highest level of education, income in the last month, and occupation; (ii) questions regarding the knowledge level of bioplastics, bio-based and biodegradable products; (iii) questions related to the usage and buying of bioplastics (frequency and type of products); (iv) questions regarding the concerns about bioplastics (for instance, the impacts of bioplastics on human and environmental health); and finally, (v) a set of questions asking for the opinions of the respondents (e.g., replacing of conventional plastic, ways to encourage the use of bioplastics).

Different formats for answers were used. For instance, to gather information about the respondents’ knowledge of bioplastics, multiple answers were possible (the same for obstacles to buying, the modalities of bioplastics, and the drivers to buy). To understand the frequency of certain behaviors, a 4-point scale was used (regularly, sometimes, rarely, and never). To identify and mitigate language and understanding problems, a pre-test of the questionnaire involving a sub-set of the respondents was carried out. Then the questions and the measurement scales were rearranged to make them clear and easy to understand.

Using the *LimeSurvey* platform, the questionnaire was made available electronically in several countries for five months (from October 2020 to February 2021). A total of 384 questionnaires were collected, and the data were statistically analyzed and interpreted using SPSS V.26. Thus, a set of statistical analyses like frequency and descriptive analysis was used to understand user attitudes and concerns about bioplastics. In addition, Chi-square tests were also used to assess the relations between independent variables such as educational level, age, and gender, considering the variables in the nominal scale (categorical) as adopted in previous studies [27]. The Chi-square test was applied to establish relations between the knowledge about bioplastics and the educational level of participants and assess the relations between the use of bioplastics and educational level and gender. Note that the statistical inference was performed, considering a significance level of 5% for all tests.

## 4. Results and discussion

### 4.1 Demographic characteristics

**Fig 1:**
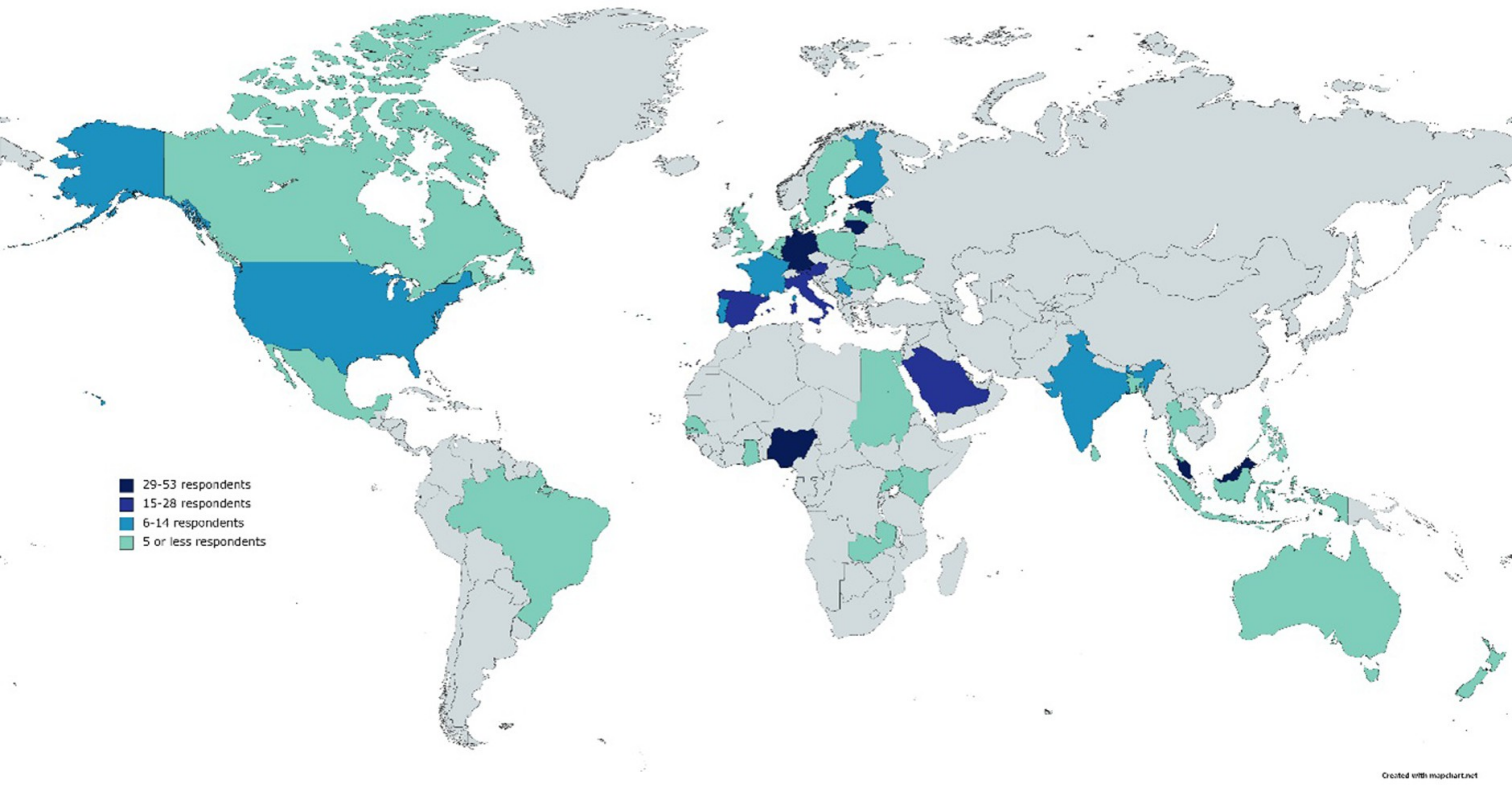
Survey participating countries and number of respondents.

A total of 384 participants from 42 countries completed the online survey from October 2020 to February 2021. Most of the respondents were from Malaysia [53], Estonia [43], Nigeria [34], Lithuania [29], and Germany [29]. Fig 1 gives a broad breakdown of the countries that participated in the study and their color gradation according to the number of participants. According to the regional distribution of survey participants, most of them were from Europe (59.6%), then from Asia (23.2%), Africa (10.7%), North America (3.9%), Latin America (2.3%), and Oceania (0.3%). The gender characteristics of the respondents were rather balanced: 47.9% of males and 51.3% of females, while 0.5% preferred not to disclose their gender. Most of the participants (33.9%) were between the ages of 26–35, followed by 28.5% with the age of 36–45. The third group (18.5%) was composed of, participants within the 46–59 age bracket. The participation of the young generation between the ages of 18–25 years was 13.3%, and that of older people (60+ years) was 5.5%.

Regarding the educational level of respondents, 40.4% had a master level degree, 27.1% had a Ph.D. degree, 21.1% had a bachelor’s degree, and 9.4% had a high school level education. The majority of the respondents (58%) were employed in private and public sectors. Professional workers formed 16.1%, respondents working in the business and academia sector formed 12.8% and 5.2%, respectively. Students formed 5.2% of the respondents and unemployed people corresponded to 0.8% of the respondents. In addition, 1.9% of participants mentioned the other occupancy regarding their working field. The statistical significances between gender, educational level, and age were assessed using the Chi-Square test. The level of significance used in the calculations was 0.05. A statistically significant relation was found between the educational level and the age of the respondents (p <0.05). However, no significant relation was estimated between gender and educational level (χ2 = 3.072; df = 4; N = 381; p = 0.546). Therefore, further analyses were performed based on the education and gender factors.

### 4.2 General knowledge of bioplastics

**Fig 2:**
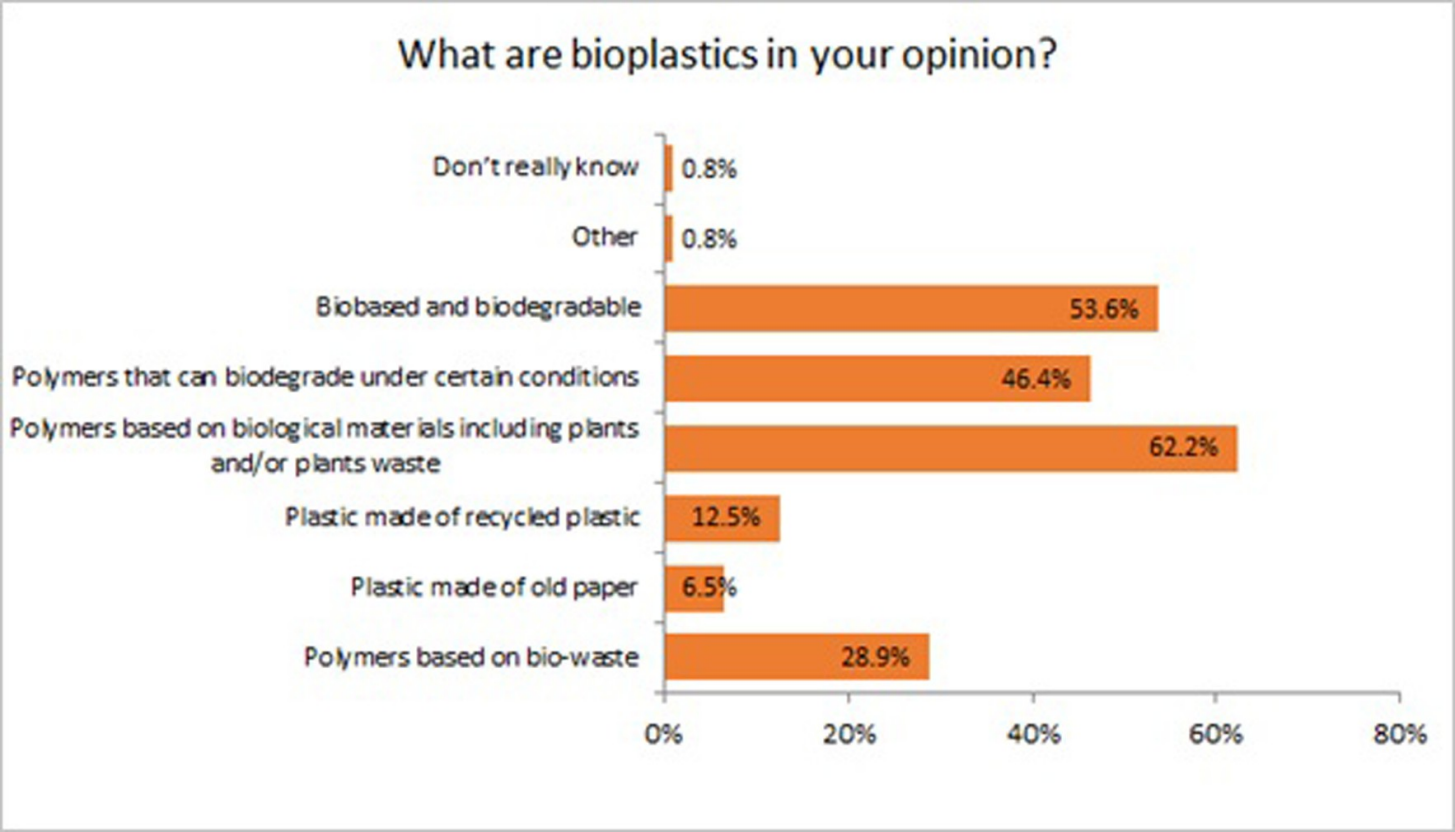
Respondents’ basic knowledge of bioplastics (n = 384).

The results of the survey show that the respondents have a good understanding of the basic concepts regarding bioplastics. The majority of respondents selected at least one correct answer (multiple answers were possible) to the question: *‘What are bioplastics*?’ However, it is a complex topic to understand and refers to a plastic that is bio-based and/or biodegradable. Most respondents identified bioplastics as: ‘polymers based on biological materials including plants and plants waste’ (Fig 2). A significant relation was found between participants who selected at least this correct answer and their level of education (χ2 = 12.225; df = 4; n = 384; p = 0.0158), which refers to a better understanding of the meaning of bioplastics by people with a higher level of education. The relation between gender and knowledge of bioplastics was not found (χ2 = 0,923; df = 1; N = 381; p = 0.337). According to [34] research results among the Australian public, the knowledge of bioplastics appeared to be relatively low, which was not confirmed by the present study results. However, the survey conducted in Europe [35] showed that more than 50% of the respondents were aware of the main properties of bioplastics.

The other two questions regarding basic bioplastics knowledge were about definitions of ‘bio-based’ and ‘biodegradable’. Again, the respondents showed a good understanding of these two concepts. About 79.2% of respondents chose the most appropriate definition for bio-based products as ‘products, which consist mainly of biological products or renewable domestic agricultural materials or forestry materials. Similarly, 82.6% identified biodegradable plastics (multiple answers were possible) as ‘given the right conditions and presence of microorganisms, it will eventually break down to its basic components. These results indicate that understanding the basic concepts associated with bioplastics has a positive shift in society. However, such a good knowledge of basic concepts of bioplastics might be associated with their high level of education (88.5% of all respondents had Bachelor’s, Master’s, or Ph.D. degrees) and perhaps cannot be representative of the entire social strata in the analyzed regions.

### 4.3 Use of bioplastic products in daily activities

**Fig 3:**
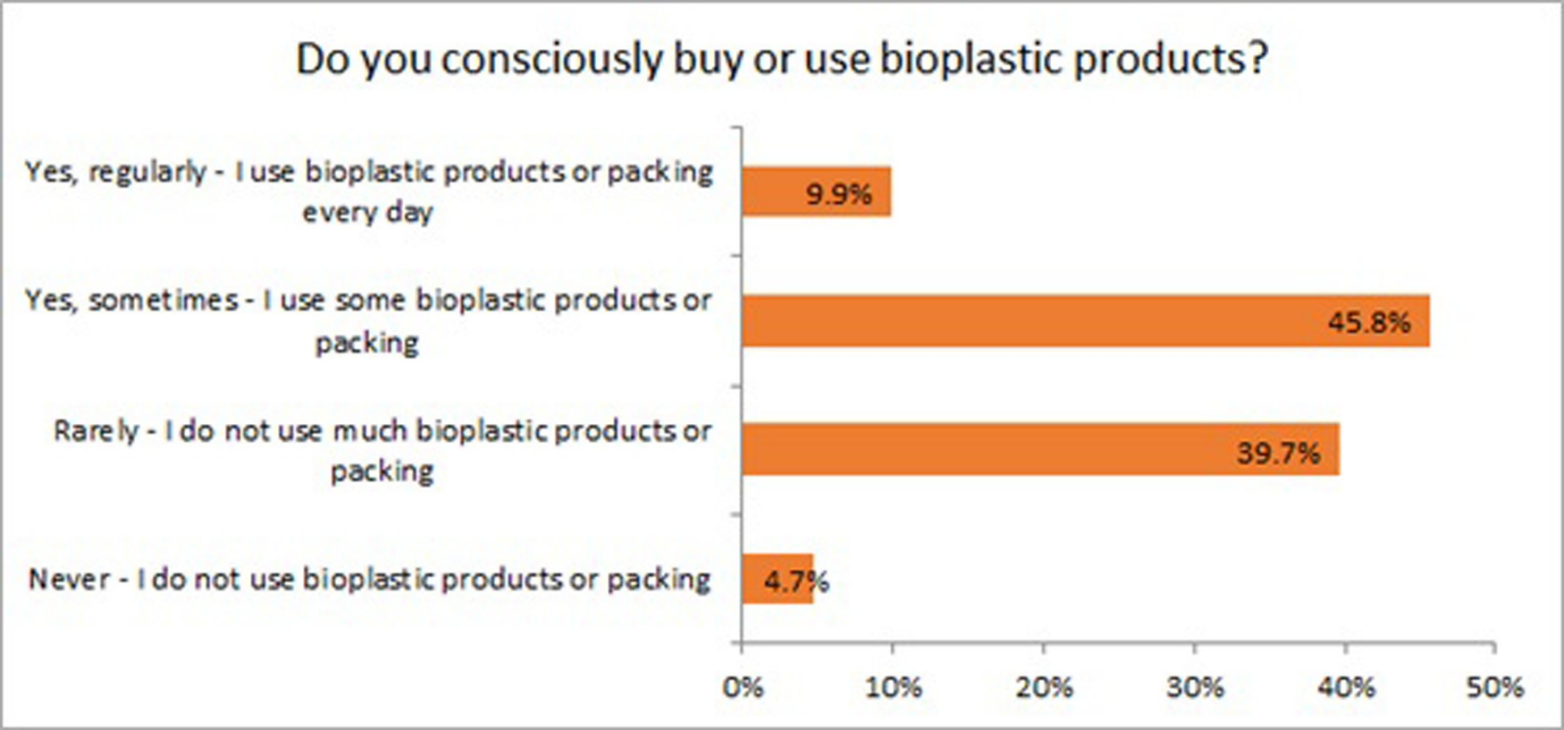
Conscious purchase and consumption of bioplastic products (n = 365).

Nowadays, conventional plastics are used regularly in daily activities throughout the world. Out of 377 respondents, about half use plastic products and plastic packaging in their everyday activities. At the same time, 42.7% of respondents mentioned that they try to avoid using plastic products and packing in some activities. In comparison, the use of bioplastic products is much more modest, only 9.9% of respondents use bioplastics in their daily life, and 45.8% of participants use bioplastic products and packaging sometimes. Also, 39.7% of participants try to avoid using bioplastic products and packing (Fig 3). This lack of adoption can be related to an unclear understanding of the properties of bioplastics, their biodegradation in the natural environment, and the association with recycling problems of bioplastics [36]. No significant associations were found between the use of bioplastics in daily activities and educational level (χ2 = 8.562; df = 8; N = 347; p = 0.381) or gender factor (χ2 = 4.163; df = 2; N = 345; p = 0.125).

**Fig 4:**
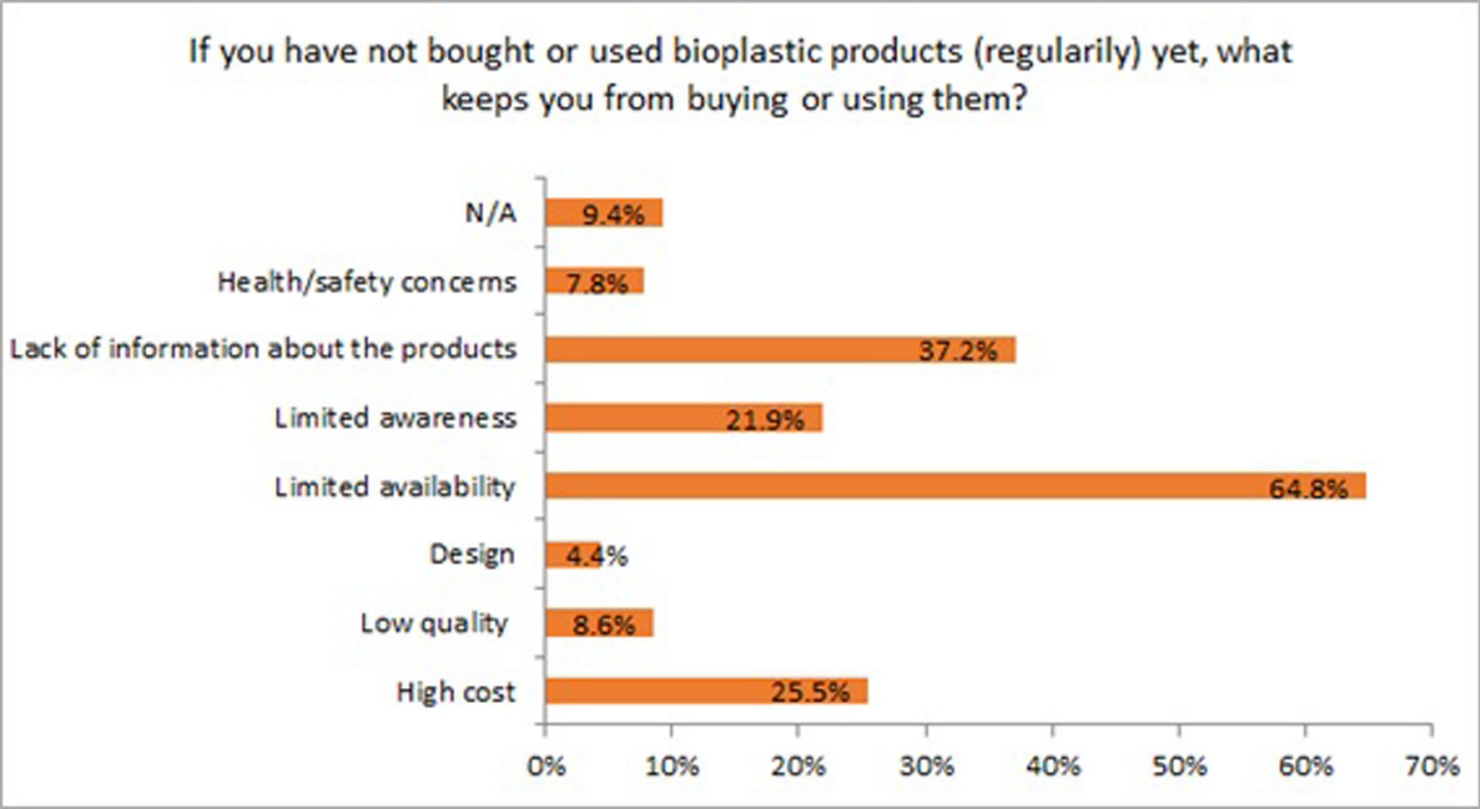
Main obstacles to buying or using bioplastic products regularly (n = 384).

The survey results indicated that the main reasons limiting the use of bioplastics in daily life activities are: limited availability (64.8%), lack of information about the products (37.2%), high cost (25.5%), and limited awareness (21.9%) (Fig 4). In addition, other obstacles were found from other studies, such as lack of or insufficient governmental support [35] and less convenience for use [34].

**Fig 5:**
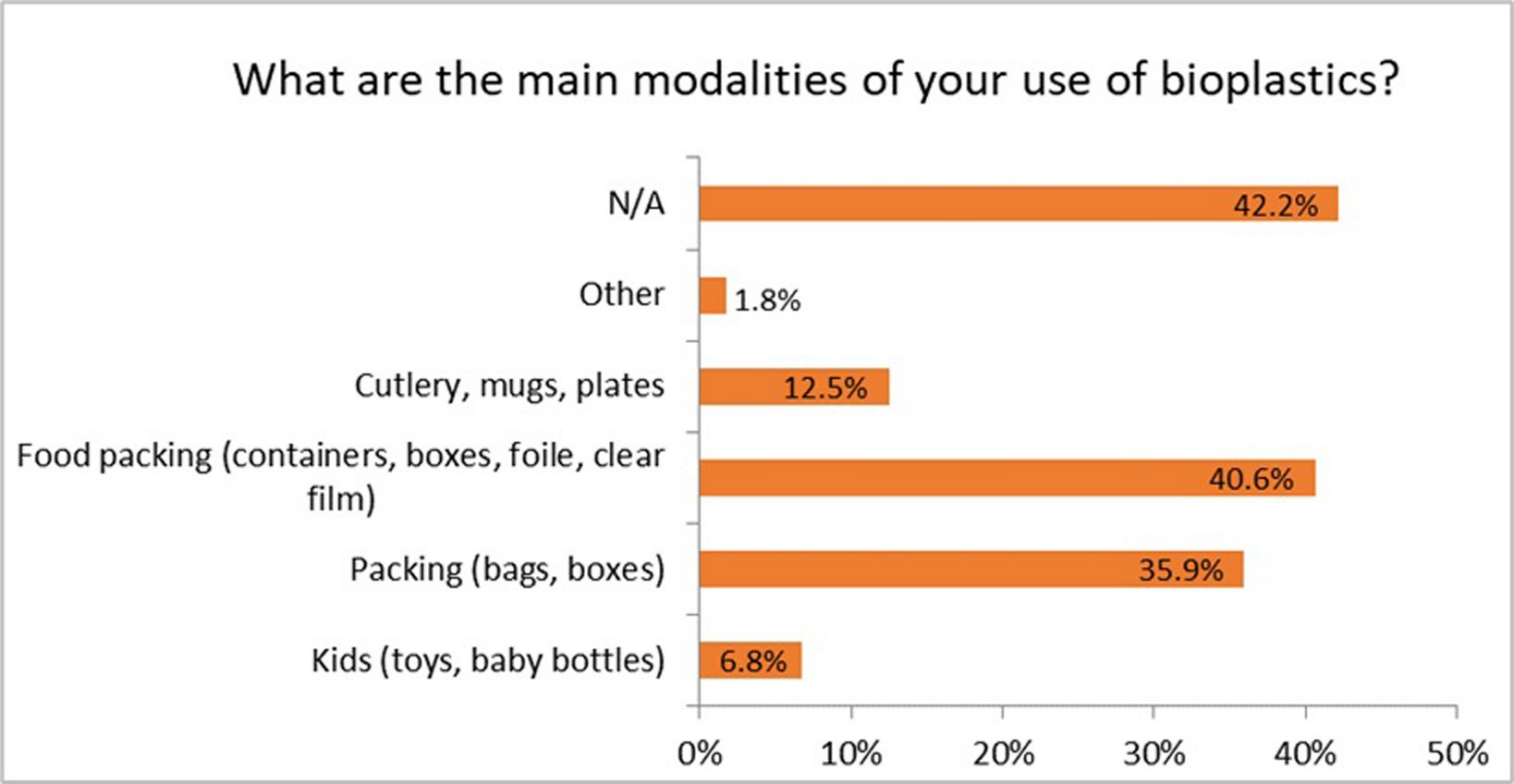
Main modalities of using bioplastics (n = 384).

The participants reported a low awareness regarding the question about the main modalities of the use of bioplastics: 42.2% of respondents selected N/A. The main uses of bioplastics were reported for food packaging (40.6%), packing (bags and boxes) (35.9%), cutlery (12.5%), and toys and baby bottles (6.8%) (Fig 5). The daily use of bioplastic products might be limited due to several factors: i) awareness of the people about bioplastics and their properties, ii) economic barriers (high price), iii) quality and design, iv) health and safety concerns. To minimize the effect of these factors, joint efforts at political and economic levels have to be implemented towards fostering a better understanding of bioplastics, their properties, use, and end-of-life options.

The use of biodegradable bioplastic for food packaging can be limited by the mechanical properties of the material in comparison with conventional plastics. As reported by Zhao et al. [37], most biodegradable bioplastics have lower tensile elongation, lower impact strength, and higher flexural modulus (lower flexibility) in comparison with conventional plastics. In addition, the thermal and chemical properties can also be improved for some biodegradable bioplastics such as PLA [38] and PHBV [39, 40].

In response to the question ‘*Would you buy bio-based and biodegradable products if they were of good quality and safe (no impact on the human health and environment)*, *but more expensive*?*’*, 90.6% of respondents answered N/A. This high level of uncertainty amongst the respondents needs further investigation to understand better people’s motivation to purchase biodegradable products. Perhaps the financial component is still crucial for the public, and people are not easily ready to invest money in the products when the information provided for consumers is insufficient for some European countries [35]. Therefore, more efforts have to be put towards increasing people’s awareness about bio-based and biodegradable products, their properties, their use, and the environmental and human health impacts.

### 4.4 Perceptions and expectations towards bioplastics

**Fig 6:**
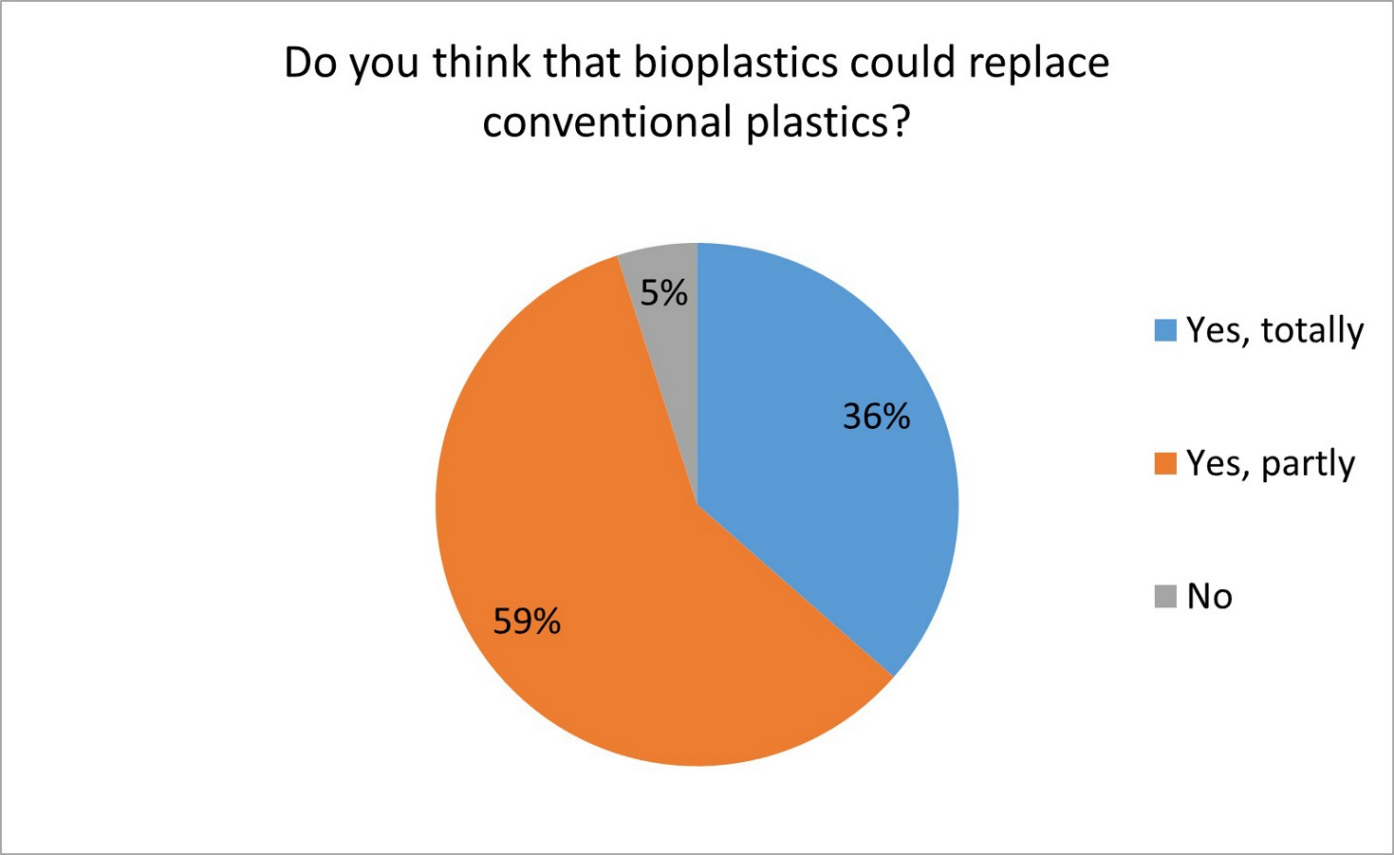
The possibility of bioplastics replacing conventional plastics (n = 384).

In general, the respondents have positive expectations regarding the future of bioplastics. In total, 95.1% of all respondents believe that bioplastics can fully or partly replace conventional plastics (Fig 6).

**Fig 7:**
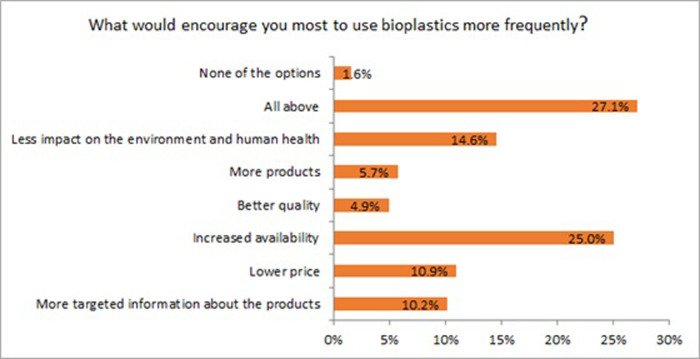
Main drivers encouraging the use of bioplastics more frequently (n = 384).

The participants also mentioned the increased availability of bioplastics as the main driver to encourage them to buy more bioplastics products (Fig 7). However, all options mentioned in this question were considered as important. These findings confirm the positive perception of bioplastics. They could be used as a message for business companies to increase the availability of bioplastics on the market to play a more prominent role in the economy in the long term. Globally, the production of bio-based and biodegradable plastic corresponds to 1% of total plastic production. However, the market for bioplastics is growing continuously [41]. Furthermore, bioplastics have great potential to switch from linear to circular economy models, considering waste as valuable material [42].

**Fig 8:**
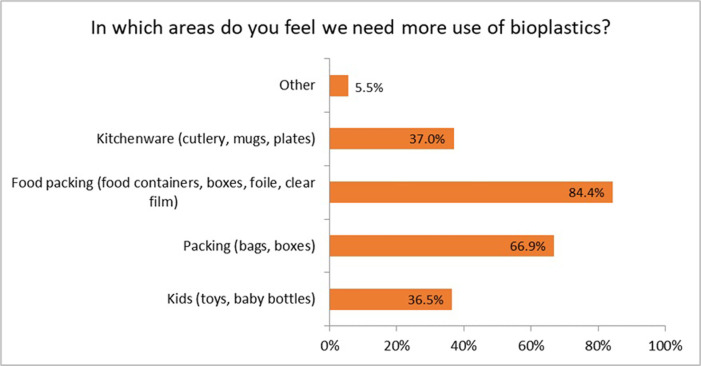
Likelihood of increasing application areas of bioplastics (n = 384).

Regarding the possible applications of bioplastics, the respondents mentioned food packaging as the main area where bioplastics should be increased (Fig 8). The biggest market segment for bioplastics is the packaging market, which includes flexible bioplastics (for food wrapping, for example) and rigid bioplastics applications [41]. Possible bioplastics applications are in general packagings, such as bags or boxes. Kitchenware, like cutlery or plates, was in third place in this regard.

These findings indicate the willingness of respondents to see more bioplastic products on the market as a feasible alternative to conventional plastics. However, even the most advanced economies are far from closing the plastic loop, and more investments are needed for green innovations [43].

The use of bioplastics instead of fossil-based plastics will help to improve the end-of-life management of plastic waste because most bioplastic materials can degrade under controlled conditions. However, there are some drawbacks found during the application of bioplastics. [44] defined four main challenging factors that limit biodegradable plastics development. The first is technological because attempts to improve the thermal stability and mechanical strength will slow down the degradation process.

The second is financial: the material price of bioplastic is higher than conventional plastic, which will impact the final market price of the product. The third is the lack of a proper waste management system, such as separate collection for bioplastics, which is essential for the application of composting and anaerobic digestion as end-of-life options. Moreover, the last point is to raise environmental awareness among the public and develop the classification system or identification codes for bioplastics. However, recent studies in Australia and Europe have shown that people’s awareness is still very low towards bioplastics and their identification [34, 35, 45].

Another point of concern that can inhibit the development and application of bioplastics is the availability of land for feedstock production. According to the European Bioplastic Association, the feedstock for bioplastic production today is about 0.02% of the global agricultural area and is not expected to grow by 2024 [41]. Secondly, the positive aspect is that the application of bioplastics in food packaging helps extend the shelf life of some products, which leads to the prevention of food waste production [46].

By this, bioplastics offer the opportunity to lower the carbon impact and reduce the dependence on non-renewable oil. However, they do not directly address the problem of plastic littering and accumulation in the environment. Therefore, informing and raising awareness among people for proper and controlled plastic management, disposal, and (organic) recycling remains a key measure to prevent plastic pollution in the environment, regardless of the plastic types [26].

## 5. Conclusions

Bioplastics are increasingly gaining momentum because of the human and ecological risks of using conventional plastics, which are non-biodegradable and highly polluting. However, the success of large-scale diffusion and adoption of bioplastics is contingent upon gauging the attitudes and addressing the concerns of bioplastics consumers. This paper presents a worldwide study on identifying and addressing consumer attitudes and concerns with bioplastics use towards improving human and ecological health. The study found that the participants have a good understanding of the links between the concepts of bioplastics and biodegradable, implying a positive view of bioplastics. Annually, millions of tons of conventional petro-plastics are incinerated or end up in landfills or water bodies, thereby destroying fauna and flora and their habitats. Conventional plastics also negatively affect human health through associated air pollution and food contamination.

The present study found that a little over half of the participants used bioplastics products regularly or sometimes, mainly for food packaging and as containers, cutlery, and toys. Thus, using bioplastics can reduce the carbon footprint by adding fewer greenhouse gases to the ecosystem. In addition, bioplastics can create more jobs than conventional plastics and foster environmental sustainability, as they are produced from plant biomass, which is renewable and compostable. Therefore, global support for bioplastics, such as banning single-use plastic bags, should be promulgated to support the bioplastics sector. This recommendation is significant because bioplastics can help switch an economy from a linear to a circular model that considers waste as valued material.

The study participants also reported that the factors militating against their use of bioplastic include the limited availability and high cost of bioplastics products, the lack of information about the products and their properties, unsatisfactory designs and quality, and little awareness about their benefits. This finding implies the need for joint efforts at political and economic levels to better understand bioplastics, their properties, environmental and health benefits, and end-of-life options. Thus, human factors can be among the significant barriers to using bioplastics.

Most respondents have positive expectations regarding the future of bioplastics to replace conventional plastics fully or partially, especially as food containers, kitchenware, and boxes and bags for packaging. They reported that the low costs and the increased availability of bioplastic products on the market are likely to be the main drivers for their wide-scale adoption. However, nine out of ten participants are unsure whether they would buy bio-based and biodegradable products if they are expensive. In contrast, they seem to appreciate the value of good quality and safety. This high level of uncertainty is present even though two-thirds of the study participants had graduate-level education. Relevant policies are therefore needed to encourage investments in large-scale manufacture and market uptake of bioplastics.

This paper has some implications to consider. Firstly, it shows the elements that constrain the willingness of some consumers to engage in the purchase of bioplastics. Secondly, matters related to feedstock production are also potential bottlenecks and should be addressed. Finally, the paper shows the need to take into account the issues related to littering: even bioplastics use can be associated with littering, and this should be addressed by, for instance, emphasizing multiple-use plastic as opposed to single-use materials.

This study has a limitation because regions such as East Asia and South America were underrepresented. Also, participation in the study was limited to people who speak English and was possible for a limited period. Nonetheless, the inputs provided by the sample of 384 participants from 42 countries provide some useful insights into how bioplastics products are perceived. The study provides evidence on consumer attitudes and concerns with using bioplastics, which is an important first step in developing appropriate policies and programs towards adopting bioplastics products, hence realizing their environmental and economic benefits. Bioplastics can effectively help achieve SDG 12, emphasizing the urgency to transition to sustainable consumption and production systems worldwide.

## Supporting information

S1 File (PDF)
