# Supplementary material for: Consumer attitudes and concerns with bioplastics use: An international study
Source: PLoS One. 2022 Apr 27;17(4):e0266918. doi: 10.1371/journal.pone.0266918 (PMC9045599; doi:10.1371/journal.pone.0266918)
Supplement: S1 File — (PDF) [file pone.0266918.s001.pdf]

Consumer Attitudes and Concerns on Bioplastics Use: towards improved human and environmental health

Thank you for considering taking part in our survey. The main aim of this survey is to assess the perceptions and concerns with regard to bioplastics among consumers and their knowledge level. This survey will take you no longer than 10 minutes to complete. If you wish to receive a copy of the results or more details about this project, please contact us: bioplastics@ls.haw-hamburg.de.

There are 19 questions in this survey.

Consent

Consent Survey Participation

I consent to participate in this survey. I understand my participation is completely voluntary and I can withdraw my consent at any time, without penalty or consequence. I further grant permission for the data generated from this survey to be used in the BIO-PLASTICS EUROPE project activities and scientific publication on the topic of consumers' perceptions and concerns with regards to bio-plastics. I have read and agreed to the privacy policy of Lime Survey (available at https://www.limesurvey.org/en/privacy-policy (https://haw-mailer.haw-hamburg.de/owa/redir.aspx?C=vx__DI4_Cozf-qQHqiAofCcVPeNMLX6W0ZjHZffzQTujpmPnOGrYCA..&URL=https%3a%2f%2fwww.limesurvey.org%2fen%2fprivacy-policy)).

*

❶ Choose one of the following answers

Please choose **only one** of the following:

◯ I agree

Demographic Data

Country of residence *

Please write your answer here:

____

Gender *

❶ Choose one of the following answers

Please choose **only one** of the following:

◯ Female

◯ Male

◯ Other

◯ No answer

Age Group (years) *

Choose one of the following answers
Please choose **only one** of the following:

- 18-25
- 26-35
- 36-45
- 46-59
- 60+
- No answer

Highest degree or level of education *

Choose one of the following answers
Please choose **only one** of the following:

- High school or less
- Bachelor´s degree
- Master´s degree
- PhD
- No answer

Occupation *

Choose one of the following answers
Please choose **only one** of the following:

- Business sector
- Public sector
- Private sector
- Professional work
- No answer
- Other

Knowledge Level

What are bioplastics in your opinion? *

Check all that apply
Please choose **all** that apply:

- [ ] Polymers based on bio-waste
- [ ] Plastic made of old paper
- [ ] Plastic made of recycled plastic
- [ ] Polymers based on biological materials including plants and/or plants waste
- [ ] Polymers that can biodegrade under certain conditions
- [ ] Biobased and biodegradable
- [ ] Other: ____________

What definition of "bio-based" products do you feel is the most appropriate one? *

Choose one of the following answers
Please choose **only one** of the following:

- ○ Products, which consist mainly of biological products or renewable domestic agricultural materials or forestry materials
- ○ Products, which consist entirely of recycled plastic bottles
- ○ Products, which consist mainly of bio-waste and if applicable paper waste
- ○ Products, which consist entirely of recycled textiles
- ○ Other ____________

What does the term "biodegradable" mean?

Check all that apply
Please choose **all** that apply:

- [ ] If something is biodegradable, then, given the right conditions and presence of microorganisms, it will eventually break down to its basic components
- [ ] If something is biodegradable, then, it can be burned without harming the environment
- [ ] If something is biodegradable, then, it can be thrown in the ocean and will do no harm to the ecosystem
- [ ] If something is biodegradable, then, it can easily be broken down in its basic components and be reused (recycled)
- [ ] Other: ____________

Are biobased products currently available on the market for consumer usage?

❗ Choose one of the following answers
Please choose **only one** of the following:

- ◯ Yes, widely
- ◯ Yes, limited
- ◯ No, still in development
- ◯ No, they are not

Usage and Attitudes with regard to Bioplastics

How often do you use conventional plastics?

❗ Choose one of the following answers
Please choose **only one** of the following:

- ◯ Regularly **-** i use plastic products or plastic packing every day
- ◯ Sometimes **-** i have been trying to avoid plastic products and packing
- ◯ Rarely **-**i do not use much plastic products or packing
- ◯ Never **-** i do not use plastic products or packing

Do you consicously buy or use bioplastic products?

❗ Choose one of the following answers
Please choose **only one** of the following:

- ◯ Yes, regularly **-** i use bioplastic products or plastic packing every day
- ◯ Yes, sometimes **-** i use some bioplastic products and packing
- ◯ Rarely **-** i do not use much bioplastic products or packing
- ◯ Never **-** i do not use bioplastic products or packing

Usage and Attitudes with regard to Bioplastics

If you use it sometimes or regularily, what are the main modalities of your use of bioplastics? *

Only answer this question if the following conditions are met:
Answer was 'Yes, sometimes - i use some bioplastic products and packing' *or* 'Yes, regularly - i use bioplastic products or plastic packing every day' at question '12 [UAb]' (Do you consicously buy or use bioplastic products?)

❗ Check all that apply
Please choose **all** that apply:

- ☐ Kids (toys, baby bottles)
- ☐ Packing (bags, boxes)
- ☐ Food packing (food containers, boxes, foile, clear film)
- ☐ Kitchenware (cutlery, mugs, plates)
- ☐ Other: ____________

Usage and Attitudes with regard to Bioplastics

Would you buy bio-based and biodegradable products if they were of good quality and safe (no impact on the human health and environment) but more expensive?

Only answer this question if the following conditions are met:
Answer was 'Never - i do not use bioplastic products or packing' at question '12 [UAb]' (Do you consicously buy or use bioplastic products?)

❗ Choose one of the following answers
Please choose **only one** of the following:

- ○ Yes
- ○ No

Concerns

If you have not bought or used bioplastic products (regularily) yet, what keeps you from buying or using them? *

Only answer this question if the following conditions are met:
Answer was 'Never - i do not use bioplastic products or packing' *or* 'Rarely - i do not use much bioplastic products or packing ' *or* 'Yes, sometimes - i use some bioplastic products and packing' at question '12 [UAb]' (Do you consicously buy or use bioplastic products?)

❗ Check all that apply
Please choose **all** that apply:

- ☐ High cost
- ☐ Low quality
- ☐ Design
- ☐ Limited availability
- ☐ Limited awareness
- ☐ Lack of information about the products
- ☐ Health/safety concerns

Health Safety

If you have any health/safety concerns with regard to bioplastics, which statement fits the best?

Only answer this question if the following conditions are met:
Answer was 'Health/safety concerns' at question '15 [Ca]' (If you have not bought or used bioplastic products (regularily) yet, what keeps you from buying or using them?)

❗ Choose one of the following answers
Please choose **only one** of the following:

- ◯ I am concerned about the more toxic components compared to those in conventional plastic products
- ◯ I am concerned about the bpa, which mimics hormone oestrogen and can cause long term effects on the human body
- ◯ I am concerned, because long term studies are not available yet
- ◯ I am concerned, because the degradation of bio-based plastics result in tiny plastic pieces that have similar characteristics and effects on health as regular micro plastic
- ◯ Other ________

Final Questions

Do you think that bioplastics could replace conventional plastics? *

Choose one of the following answers
Please choose **only one** of the following:

- ◯ Yes, totally
- ◯ Yes, partly
- ◯ No

What would encourage you most to use bioplastics more frequently? *

Choose one of the following answers
Please choose **only one** of the following:

- ◯ More targeted information about the products
- ◯ Lower price
- ◯ Increased availability
- ◯ Better quality
- ◯ More products
- ◯ Less impact on the environment and human health
- ◯ All above
- ◯ None of the options

In which areas do you feel we need more use of bioplastics? *

Check all that apply
Please choose **all** that apply:

- ☐ Kids (toys, baby bottles)
- ☐ Packing (bags, boxes)
- ☐ Food packing (food containers, boxes, foile, clear film)
- ☐ Kitchenware (cutlery, mugs, plates)
- ☐ Other: ____________

The project "Bio-Plastics Europe" https://bioplasticseurope.eu/ has created the European Bio-Plastics Research Network. If you wish to join it, please contact the project team at: bioplastics@ls.haw-hamburg.de. Thank you for taking part in the survey!

Submit your survey.
Thank you for completing this survey.
